# A unified model for cross-modal plasticity and skill acquisition

**DOI:** 10.3389/fnins.2024.1334283

**Published:** 2024-02-07

**Authors:** Woon Ju Park, Ione Fine

**Affiliations:** ^1^Department of Psychology, University of Washington, Seattle, WA, United States; ^2^Center for Human Neuroscience, University of Washington, Seattle, WA, United States

**Keywords:** skill acquisition, development, plasticity, visual cortex, cross-modal, blindness, neuronal recycling, cultural recycling

## Abstract

Historically, cross-modal plasticity following early blindness has been largely studied in the context of visual deprivation. However, more recently, there has been a shift in focus towards understanding cross-modal plasticity from the perspective of skill acquisition: the striking plasticity observed in early blind individuals reflects the extraordinary perceptual and cognitive challenges they solve. Here, inspired by two seminal papers on skill learning (the “cortical recycling” theory) and cross-modal plasticity (the “metamodal” hypothesis) respectively, we present a unified hypothesis of cortical specialization that describes how shared functional, algorithmic, and structural constraints might mediate both types of plasticity.

## 1 Introduction

Perhaps the capacity that most differentiates humans from other animals is our cognitive plasticity, especially during development. The ability to learn a wide array of novel skills, from reading to calculus, continues over a prolonged developmental period that lasts almost two decades. Many of these tasks involve the development of novel specialized areas in the brain or “hyper-abilities” within existing areas.

This flexibility may underlie the remarkable ability of blind individuals to navigate in a sighted world. From the first moment they interact with the world, early blind individuals learn different skills. A sighted baby recognizes her father’s face as he approaches the crib, a blind baby recognizes the sound of his footsteps. A sighted toddler looks towards a dog to attract her parent’s shared attention while a curious blind child pulls her parent’s hand in the direction of the barking.

As a result, the experience of early blindness provides one of the most dramatic examples of plasticity that can be observed in the human brain: large regions of cortex that are normally predominantly driven by visual input respond to a wide variety of auditory and tactile tasks (Fine and Park, 2018). Until fairly recently, this *cross-modal plasticity* was primarily studied from the perspective of sensory deprivation. It was assumed that the primary factor driving cortical organization was the loss of vision, and rats dark-reared in a deprived environment were considered a close model system for early blind humans. The last decade or so has seen a shift in perspective: a recognition that much of cross-modal plasticity may not be due to deprivation *per se*, but rather may reflect the strikingly different perceptual and cognitive demands posed by blindness.

According to this framework, cross-modal plasticity in early blind individuals is not sui generis – a unique form of plasticity - but rather utilizes the same mechanisms and operates under the same constraints as the plasticity that underlies the acquisition of other specialized skills that require extensive experience in development to reach full fluency (such as reading, arithmetic, or playing the flute). Here, we propose a unified model of plasticity which brings together theories from both the sensory deprivation and skill acquisition literature: we propose that the same structural and functional constraints guide cross-modal plasticity as a result of sensory loss and the development of specialized cortical areas for novel skills.

One important reason for suspecting that cross-modal plasticity and skill learning share common mechanisms is that they seem to have similar critical periods. The critical period for cross-modal plasticity does not match the sensitive period for visual development (under 5 years of age) but rather seems to match the sensitive period seen for complex skill development, which extends well into adolescence (Sadato et al., 2002).

Our postulates are essentially a synthesis of two papers that discuss cross-modal plasticity and complex skill learning, respectively. In 2001, in *“The metamodal organization of the brain”*, Pascual Leone and Hamilton (Pascual-Leone and Hamilton, 2001) proposed that the traditional view of the brain as being organized in parallel unimodal sensory systems was incorrect. Instead, cortical organization *“might actually represent a metamodal structure organized as operators that execute a given function or computation regardless of sensory input modality.”* For example, according to this model, the role of hMT+, an area primarily specialized for processing visual motion in sighted individuals, is to track the motion of objects and send this information to other cortical areas. In the absence of vision, the same functional goal can be achieved using information from audition or touch.

Just a few years later, in *“Cultural recycling of cortical maps”*, Dehaene and Cohen (Dehaene, 2005; Dehaene and Cohen, 2007 for a review of related theories, see Hernandez et al., 2019) proposed that skill learning leads to specialized cortical regions (such as the visual word form area, or areas involved in numerosity) as a consequence of “neuronal recycling” – that is, the development of cortical areas for culturally novel skills takes place by repurposing evolutionarily older circuits. They further argued that, because pre-existing neural constraints persist, novel specialized brain areas are tightly constrained by prior evolution and brain organization.

Merging these two papers leads to three postulates on how cortical specialization is modulated in both sensory loss and skill learning:

- Postulate I: Cortical specialization retains an area’s functional goals.
- Postulate II: Cortical specialization is shaped by feedforward and feedback inputs.
- Postulate III: Cortical specialization is constrained by neuroanatomical architecture.

Each of these postulates maps closely onto one of the levels of analysis described by Marr (2010). Postulate I is focused on Marr’s level 1: *What is the functional goal that this area serves?* Postulate 2 maps onto level 2: *What are the information-processing algorithms that support this specialization?* Postulate III focusses on Marr’s third level of analysis: *How are these algorithms implemented and mapped to the neural architectures of the brain?*

## 2 Postulate I: Cortical specialization retains an area’s functional goals

A central prediction from both metamodal and cortical recycling theories is that each cortical area serves a functional goal, and that the development of cross-modal plasticity or novel specialized areas takes advantage of functional homologies between the original purpose of an area and the “novel” specialization (Pascual-Leone and Hamilton, 2001; Dehaene and Cohen, 2007).

In the skill acquisition literature, this prediction has been tested in the context of reading by examining the emergence of cortical areas selective to culturally novel visual features (e.g., words) as people acquire reading skills. One early study found that the area in left ventral temporal cortex typically associated with visual word processing in literate adults is responsive to faces in illiterate adults, while the representation for faces in literate adults was largely confined to the right hemisphere (Dehaene et al., 2010). This finding has been interpreted as evidence for the idea that reading selectivity develops within a cortical area whose original function is to process local combinations of visual features (Dehaene and Cohen, 2007). This has been more recently corroborated by longitudinal fMRI studies, which observed the development of selectivity to visual words in areas that were previously selective to images of tools (Dehaene-Lambertz et al., 2018) and limbs (Nordt et al., 2021) in school-aged children as their reading skills develop.

Further evidence that reading areas inherit the functional goal of analyzing local combinations of visual features comes from recent work using deep convolutional neural networks, which showed that the processing of visual words relies on reusing visual feature templates learned from more general object categories (Janini et al., 2022). Specifically, visual features modeled from various object categories could predict perceptual similarity of letters better than those learned from letter categories only, consistent with the notion that letter recognition relies on recycling cortical regions that previously represented other object categories.

Interestingly, the specific regions that are “recycled” for reading seem to depend on the demands of the specific language(s) that are learned. An fMRI study of reading in bilingual readers found that, in English-Chinese bilinguals, the representations for Chinese letters developed in patches separate from that for English, co-localized within regions that also showed high specificity to faces. One explanation is that Chinese letters require a high degree of sensitivity to the global configuration of visual features, so specialization for Chinese requires additional recruitment of face-selective areas whose original function was to analyze global configurations (Zhan et al., 2023). In English-French bilinguals, in contrast, there was near complete overlap between the cortical representation of the two languages.

Analogously, the cross–modal responses found in early blind individuals show strong homologies with the original visual function across many of the specialized visual cortical areas. Visual motion area hMT+ responds to auditory motion (Saenz et al., 2008; Wolbers et al., 2011; Jiang et al., 2014), the visual word form area responds to Braille (Sadato et al., 1996, 1998; Burton et al., 2002), and the fusiform gyrus, typically associated with face processing, responds to voices and tactile faces in early blind individuals (Hölig et al., 2014; Ratan Murty et al., 2020). Furthermore, category selectivity for faces, scenes, body, and objects is present in higher visual areas of congenitally blind individuals (van den Hurk et al., 2017).

However, the obvious parallels in function described above may mask significant complexities. As described above, it is well established that hMT+ shows robust responses to auditory motion in early blind individuals (Saenz et al., 2008; Wolbers et al., 2011; Jiang et al., 2014), and this cross-modal homology has historically been described in terms of hMT+ having the functional role of continuously tracking object motion over space and time (Movshon et al., 1985). However, recent work has shown that both sighted and early blind individuals infer auditory motion from the sound locations at sound onsets and offsets (Park and Fine, 2023). One possibility is that the recruitment of hMT+ does not reflect the spatiotemporal computations of neurons in this region, but rather its role as “hub”: hMT+ not only computes object motion information, but is also the main source of object motion information for numerous cortical areas responsible for navigating and interacting with the 3D world. Indeed, hMT+ has projections (Abe et al., 2018) to a variety of sensorimotor areas including parietal V6 and V6A, AIP, MIP, LIP frontal A4ab, prefrontal A8aV and A8C (Grefkes and Fink, 2005; Gamberini et al., 2011; Pitzalis et al., 2013; Nelissen et al., 2017).

One apparent difficulty with Postulate I is the auditory and tactile responses found for a wide variety of stimuli within early retinotopic visual cortex in congenitally blind individuals (see Bedny, 2017, for a comprehensive review and alternative explanation of these findings). Numerous studies have observed cross-modal responses in early retinotopic areas that are modulated by higher-level cognitive tasks such as working memory (Rao and Ballard, 1999), grammatical complexity (Lane et al., 2015) and mathematics (Bedny, 2017). On the face of it these tasks have no functional overlap with the functional role of early retinotopic areas – carrying information about the presence and location of visual features. However, V1 also possesses circuitry and plasticity that support more complex functions (Gavornik and Bear, 2014a), such as information about the learned timing of reward in relation to sensory input (Chubykin et al., 2013), stimulus familiarity (Cooke and Bear, 2010), stimulus predictability (Rao and Ballard, 1999; Uran et al., 2022), and spatiotemporal sequence learning (Xu et al., 2012; Gavornik and Bear, 2014b). It’s easy to imagine how these more abstract computational roles might be recycled to support working memory, language processing, or mathematics.

In summary, both the skill-learning literature and the cross-modal literature is consistent with the idea that cortical specialization retains an area’s functional goals, with the caveat that an area may have more than one functional role, and those roles cannot always be defined intuitively.

## 3 Postulate II: Cortical specialization is shaped by feedforward and feedback inputs

Both “neural recycling” for skill learning and the metamodal theory of cross-modal plasticity rely on the concept that cortical areas (or neural “modules”, Jacobs, 1999) “compete for functional role”. According to one classic model, the “mixture of experts” Jacobs, 1999, each brain region has specific structural properties (e.g., the pattern of internal neuronal connectivity or the pattern of input and output pathways), that predispose them to be well or poorly suited to different types of functions. During learning, a “gating network” provides feedback that adjusts the weights placed on the output of each “module” based on the area’s performance on the task. As a result, over development, the area that is structurally or functionally better equipped to perform that particular task “wins the competition” to take over a given functional role. In this model, it is also generally assumed that, while cortical areas can perform more than one task, these multiplexing capacities are not infinite (Jacobs, 1999).

This model of cortical specialization was originally used to explain cerebral lateralization for language including anomalous dominance (Bever, 1980; Geschwind and Galaburda, 1985; Kosslyn, 1987). In language development, there is evidence that language network is bilateral in young children (ages 4 through 6), which becomes lateralized to the left hemisphere in later years (Olulade et al., 2020). However, in some individuals (often left-handers), language remains bilateral or can even be right lateralized. Consistent with the idea that areas are limited in their multiplexing abilities, the shift of language from the left to the right hemisphere is accompanied by a complementary shift in responses to a task that is thought to target spatial attention, from the right to left hemisphere (Cai et al., 2013).

Cross-modal responses observed in many of the visual areas in early blind individuals can similarly be interpreted under this framework. In the absence of vision, the visual motion processing area, hMT+, takes over the functional role of processing auditory object motion through competitive interactions with other brain regions, because hMT+ has innate properties that generalize to auditory motion processing. Consistent with the idea of competition between areas for cortical role, evidence suggests that the recruitment of hMT+ for auditory motion processing in early blind individuals is accompanied by a *loss* of selectivity to auditory motion in the right planum temporale (Dormal et al., 2016; Jiang et al., 2016).

However, functional specialization within cortex is also heavily shaped by feedforward processes, such as thalamic inputs to cortex. Elimination of all thalamic projections early in development results in primary visual and somatosensory areas losing their sharp boundaries with adjacent areas (Vue et al., 2013). Loss of the retina or decreasing the size of the dorsal lateral geniculate nucleus during prenatal development (before E80) results in a reduction of the size of the primary visual area (striate cortex, “area 17”) and changes in cortical folding (Dehay et al., 1989; Reillo et al., 2011; Andelin et al., 2019). Conversely, experimentally increasing the size of the dorsal lateral geniculate nucleus via genetic manipulation in mice results in a corresponding expansion in the size of the primary visual cortex (Vue et al., 2013).

Analogously, changes in cortical specification reciprocally alter thalamic responses via feedback (Antón-Bolaños et al., 2018). The cortex contains area-specific molecular features that are prenatally specified and exist independently of thalamic input (Grove and Fukuchi-Shimogori, 2003). These intrinsic factors help guide thalamocortical axons towards their cortical destination, and disruption of the cortical map engages a top-down process that influences thalamic organization (Antón-Bolaños et al., 2018). For example, the selective loss of specific barrel cortex representations in cortical somatosensory cortex (via deletion of Pax 6) are retrogradely transferred to the thalamus resulting in a thalamic re-patterning that mirrors the aberrant body map in cortical S1 (Zembrzycki et al., 2013). Similarly, monocular deprivation during development strongly shifts the eye-specific responsiveness of not only V1 but also thalamic neurons during the critical period in young mice, and it is thought that this shift in eye-dominance is due to reciprocal interactions between the two areas (Sommeijer et al., 2017).

Thus, it is plausible that impact of skill learning and cross-modal plasticity on cortical specialization are mediated by similar mechanisms of feedforward and feedback plasticity. Unfortunately, our understanding of how these mechanisms operate, within both skill learning and cross-modal plasticity, remains surprisingly limited.

## 4 Postulate III: Cortical specialization is constrained by neuroanatomical architecture

Classic cortical recycling theory posits that cultural inventions, such as reading or arithmetic, invade evolutionarily older brain circuits and inherit many of their structural constraints (Dehaene and Cohen, 2007).

The gross white matter tracts of the brain are determined prenatally, prior to the onset of visual experience, and are largely guided by molecular signaling (see Park and Fine, 2020, for a review). It has long been known that re-routing white matter pathways is sufficient to change functional selectivity – the re-routing of visual thalamic input to auditory cortex early in development results in visual functional responses in that area (Sur et al., 1988; Pallas et al., 1990).

White matter pathways have similarly been shown to determine areal specialization in humans: the individual location of specialized visual areas can be predicted by the projection zones of anatomical connectivity and cytoarchitecture (Saygin et al., 2012, 2015, 2016; Grotheer et al., 2021; Huber et al., 2021). For example, in children, cytoarchitectonic area (Fusiform Gyrus 4) has a distinct cortico-cortico profile of white matter connections that precedes experience with words. As functional specialization occurs, this cytoarchitectonic area subdivides into word and face subregions, which in turn causes some refinement of white matter tracts (Kubota et al., 2023).

Additional evidence that macroscale white matter anatomy determines function rather than vice versa comes from the surprising *lack* of white matter plasticity found as a result of early or congenital blindness. Although there is clear atrophy of connections between retina and cortex (Rokem et al., 2017), the retinotopic organization of early visual areas persists (Bock et al., 2015; Striem-Amit et al., 2015) and numerous studies have failed to find convincing evidence for major alterations in cortico-cortico white matter connections (Bock and Fine, 2014). Indeed, the specialized cross-modal responses found in early blind individuals develop in a topographically predictable manner that closely resembles the cortical functional specializations observed in sighted individuals (van den Hurk et al., 2017).

These anatomical constraints also operate at a smaller scale: functional properties can be predicted from both mesoscale (Moeller et al., 2008) and microscale (Briggman et al., 2011; Glickfeld et al., 2013) patterns of input and output projections. For example, dark reared mice show preferential intracortical connections between neurons with similar functional responses – although this selectivity is weaker than in normally reared mice (Ko et al., 2014).

One possibility is that areal circuitry is designed to support a diversity of multi-neuron spike firing patterns from overlapping sets of neurons (Sadovsky and MacLean, 2014) that can be recruited for a variety of roles. Presumably cytoarchitectural regional differences in cellular composition and local microcircuitry underlie variations in the range of computations a given cortical area can efficiently support, contributing to higher-level hierarchical organization (Cadwell et al., 2019). However, little is known as yet about how anatomical microcircuitry constrains functional processing in the case of culturally novel skill learning or sensory deprivation in humans.

## 5 Conclusion

The learning of culturally novel skills and cross-modal plasticity as a result of blindness seem to be mediated by very similar constraints and mechanisms. These constraints and mechanisms are highly analogous at a functional (Postulate I) and algorithmic (Postulate II) level, and are likely to be implemented by heavily overlapping neural mechanisms (Postulate III). Is cross-modal plasticity following early blindness nothing more than skill learning?

There is no doubt that early blind individuals learn a wide variety of culturally novel skills: from Braille to echolocation. However, it is also clear that the absence of visual deprivation plays an important role. The onset of visual experience triggers a cascade of molecular and neural signaling that, over time, refines and then stabilizes functional responses along the visual hierarchy (Park and Fine, 2020). The absence of visual input during the critical period prolongs a developmental immature state of excitation and structural instability (Hensch, 2003; Takesian and Hensch, 2013). This prolonged period of heightened plasticity may facilitate the dramatic cross-modal plasticity that is observed in early blind individuals.

According to this model, cross-modal plasticity can be seen as a heightened version of skill acquisition, wherein the critical period is extended. If so, the development of functional organization, whether in the context of skill-acquisition or cross-modal plasticity, is regulated by common anatomical and functional mechanisms. Merging these two bodies of research has the potential to provide important new insights into the remarkable capacity of humans to acquire novel skills and abilities.

## Author contributions

WP: Conceptualization, Funding acquisition, Writing – original draft, Writing – review & editing. IF: Conceptualization, Funding acquisition, Writing – original draft, Writing – review & editing.
